# Development and field evaluation of a novel sugar bait device for controlling residential vector mosquitoes in Zhejiang Province, China

**DOI:** 10.3389/fvets.2024.1364740

**Published:** 2024-03-27

**Authors:** Yuyan Wu, Chuan Zhang, Wei Feng, Sanjun Fu, Weibo Dong, Jinna Wang, Qinmei Liu, Tianqi Li, Mingyu Luo, Zhenyu Gong

**Affiliations:** ^1^Zhejiang Provincial Center for Disease Control and Prevention, Hangzhou, Zhejiang, China; ^2^Fenghua District Center for Disease Control and Prevention, Ningbo, Zhejiang, China

**Keywords:** sugar bait, vector control, a novel sugar bait device, mosquito-borne diseases, dengue fever

## Abstract

**Background:**

Mosquito-borne diseases pose serious public health threats in Zhejiang Province, China, and vector control is believed to be the primary method for reducing transmission. Due to severe resistance problems, effective and sustainable methods without chemical insecticides are urgently required to control mosquito vectors. Attractive toxic sugar baits (ATSB) are newly developed methods to control mosquitoes in recent decades with the core element sugar bait, which was invented according to the sugar-feeding behavior of mosquitoes. In this study, we developed a Novel Sugar Bait Device (NSBD) trap by combining sugar bait and physical adhesive capture technology. The study aimed to evaluate the effect of the NSBD trap on controlling mosquitoes in residential environments and to identify the optimal sugar solution concentration in the sugar bait of the NSBD for real use.

**Methods:**

Four residential villages in Ningbo City with similar geographic environments and mosquito densities were selected for field trials in 2022. One village (site 1) was designated as the control group, and three villages (sites 2–4) served as the test groups to assess the effectiveness of NSBD traps with different sugar solution concentrations (6, 8, and 10%) in the sugar bait. Larval and adult mosquito densities were monitored monthly before and semi-monthly after the trials using the CDC light trap and larval pipette method.

**Results:**

Before the trials, we monitored mosquito density for 3 months to confirm the baseline mosquito density among the four sites, and no statistical differences in adult and larval mosquitoes were found (adult, *F* = 3.047, *p* > 0.05; larvae, *F* = 0.436, *p* > 0.05). After the trials, all NCBD traps effectively controlled larval and adult mosquito densities, with the highest standard decrease rates of larval and adult mosquito densities at 57.80 and 86.31%, respectively, observed in site 4. The most suitable sugar solution concentration in the sugar bait was 10%.

**Conclusion:**

NSBD traps effectively controlled mosquitoes in residential environments during field trials. Without the use of insecticides, this may be a promising choice for mosquito vector control to prevent mosquito-borne diseases.

## 1 Introduction

Mosquito-borne diseases, such as dengue fever, malaria, and Japanese encephalitis, have imposed a significant disease burden in Zhejiang Province, located on the south-eastern coast of China. Due to its suitable climate, robust tourism, and active commercial exchanges, Zhejiang has emerged as one of the provinces with the highest incidence of dengue fever. In the last century, dengue fever outbreaks occurred continuously in Hangzhou City (1928–1929, 2017), Ningbo City (1928, 2004, 2018), Yiwu City (2009), Shaoxing City (2015), and Wenzhou City (2019). According to a study, dengue fever cases were reported in 95.55% (86/90) of towns in Zhejiang province between 2015 and 2019 (1). In 2018, dengue virus genotype I was identified from *Aedes albopictus* sampled in Wenzhou City (2, 3). The Japanese encephalitis virus was detected in Zhejiang Province in 2023 via integrated vector surveillance of arthropod vectors, with a positive rate of 0.491‰ (unpublished data). Arboviral illnesses have become a public health threat in Zhejiang Province.

Mosquito control is believed to be a more feasible method for preventing mosquito-borne diseases than commercial vaccines and drugs (4, 5). However, traditional control measures rely heavily on chemical insecticides such as pyrethroids, organophosphates, and carbamates. Excessive and repeated use of chemical insecticides has caused serious drug resistance in field populations of mosquitoes in Zhejiang. According to the results of insecticide susceptibility tests conducted in 2020 via the Zhejiang integrated vector surveillance, almost all *Ae. albopictus* field populations exhibit resistance to pyrethroids (3). Knockdown resistance (kdr) mutations F1534S were detected in all *Ae. albopictus* field populations sampled from northern, southern, western, and central Zhejiang, with the highest mutation frequency at 88.37% (Yiwu city) (6). Resistance has become a major challenge for current mosquito control strategies in Zhejiang. Additionally, using large amounts of chemicals in residential environments may pollute the environment and harm human health. Thus, there is an urgent need to devise effective and environmentally friendly methods for controlling vector mosquitoes.

In contrast to traditional chemical control methods, attractive toxic sugar baits (ATSB) have been developed as a new mosquito control method. ATSB is based on the feeding behavior of mosquitoes. Adult mosquitoes feed on sugars such as nectar and honeydew as energy sources (7). Sugars and insecticides have been developed to attract and kill mosquitoes (8, 9). In 1965, Lea prepared a solution by mixing sugar bait with malathion in a laboratory and tested its effect on killing *Aedes aegypti*. An 85.2% mortality rate of the *Ae. aegypti* has been observed in the laboratory tests (7). ATSB has been proven effective in controlling adult mosquitoes (8). However, ATSB also uses chemical insecticides to kill mosquitoes, and insecticidal resistance cannot be avoided. Based on this consideration, we combined sugar baits and physical adhesive capture technology to develop a novel sugar bait device (NSBD) trap. The sugar bait used in the NSBD was identified via laboratory tests. Semi-field trials on NSBD have been conducted previously, showing that NSBD effectively killed *Ae. albopictus* and *Culex quinquefasciatus* (unpublished data). In this study, we selected four residential villages to test the effect of NSBD on controlling mosquitoes in a real environment, aiming to identify the effect of NSBD and clarify the most suitable sugar bait components for use in real residential environments. Finally, we evaluated the potential of NSBD for actual applications in controlling mosquitoes in residential environments.

## 2 Materials and methods

### 2.1 Ethics statement

This study was approved by the Ethics Committee of the Zhejiang Provincial Center for Disease Control and Prevention (approval number: 2019-048).

### 2.2 The novel sugar bait device trap

**Figure 1:**
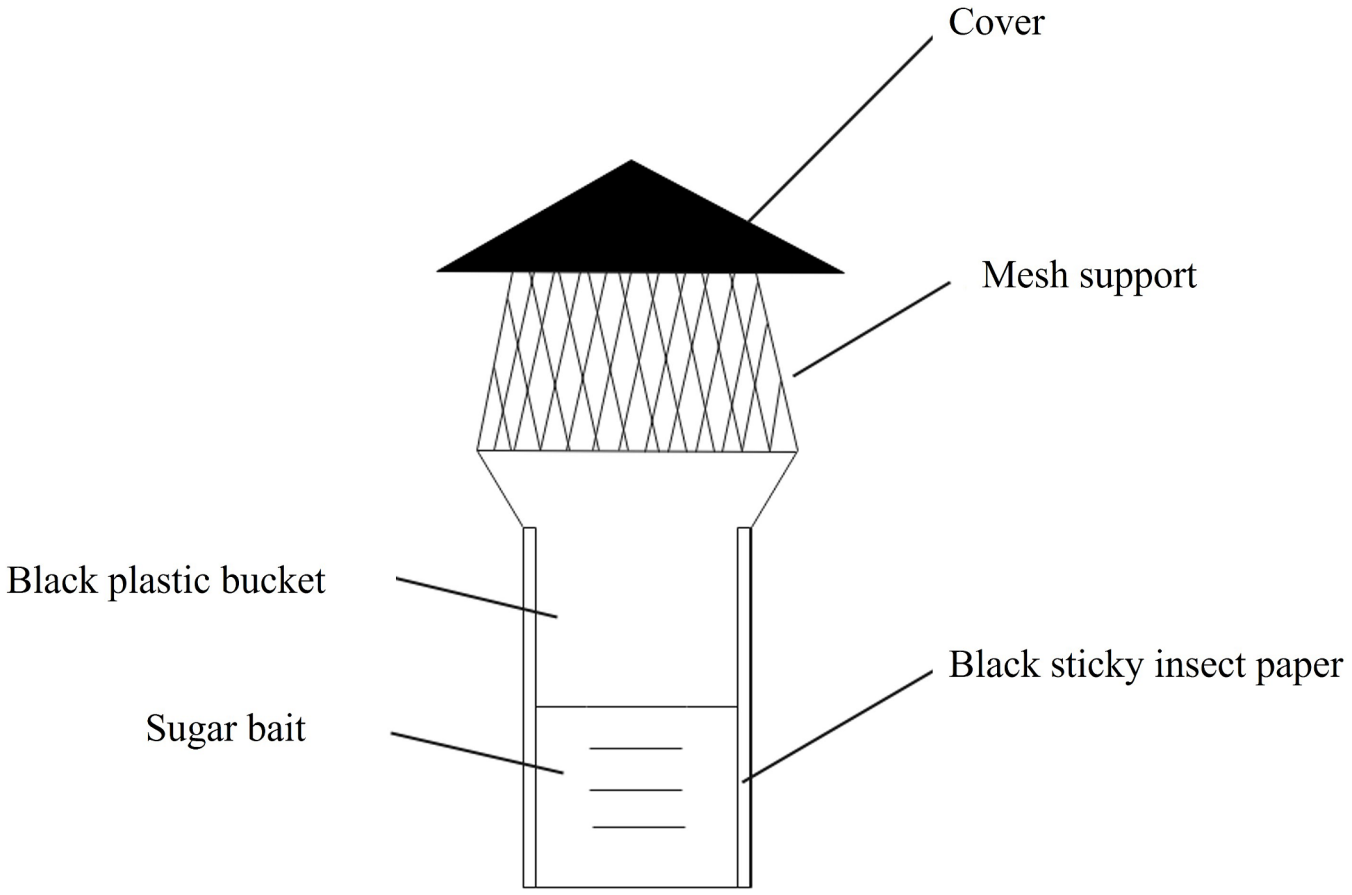
The overall schematic diagram of the novel sugar bait device (NSBD) trap.

The novel sugar bait device (NSBD) trap is shown in Figure 1. It consisted of five parts. Part 1 exhibited a cover that protected the trap from rainwater to maintain a stable sugar bait concentration. Part 2 was a mesh support with holes allowing the mosquitoes to fly into the trap. Part 3 comprised a black plastic bucket. Part 4 represented the sugar bait. Part 5 featured a black sticky insect paper adhering to the inwall of the plastic bucket for mosquitoes to land on. The sugar bait consisted of different concentrations (6–10%) of sugar solution, 1 g/L sodium benzoate, and 100 mg/L ammonium sulfate hydrochloride, and it was put inside the bucket with the volume reaching half of the bucket capacity. The relationships between the different sugar baits and NSBD traps are shown in Table 1.

**Table 1:** The geographical information for the four villages in field trials.

Sites	Coordinates	Sugar solution concentrations (glucose) in sugar bait	NSBD
Site 1	121.474812, 29.669169	Control group	None
Site 2	121.399339, 29.622876	6%	NSBD-1
Site 3	121.490556, 29.687967	8%	NSBD-2
Site 4	121.411018, 29.582785	10%	NSBD-3

### 2.3 Study sites

Field trials were conducted between July and November 2022 in Ningbo City, Zhejiang Province, China. Before the trials, we selected four villages (sites 1–4) in Ningbo with similar geographic environments (area size ranges from 4,000 to 9,000 square meters) and mosquito densities. One village was randomly chosen as the control group and named site 1, and the other three villages (sites 2–4) were designated as the test group. The villages were at least 200 m apart (Table 1). To confirm whether mosquito density was comparable among the four sites, we monitored the mosquito larvae and adults monthly from April to June 2022 before the trials began. For mosquito larvae, 30 residential yards in each site were randomly chosen and all larval breeding sites there were monitored using the larval pipette method (Container Index, CI, CI = number of containers with living mosquito larvae/number of ponding containers *100%) recommended by the national standard for surveillance methods for vector density (10). For mosquito adults, one CDC light trap was set in each site for one-overnight monitoring (female adults per trap night) according to Chinese national vector monitoring programme (11).

### 2.4 Study design

Based on previous laboratory studies and semi-field trials, we selected a sugar (glucose) concentration of 8% as the baseline. To identify the most suitable sugar bait concentration for use in real residential environments, we fluctuated a set of sugar concentrations (6, 8, and 10%) as sugar bait in NSBD traps by two percentage points. Site 1 was designated the control group, and no mosquito control measures were performed. In sites 2–4, NSBD traps were positioned on flat ground in places away from direct sunlight, rainfall, and wind, with one NSBD trap every 30 square meters. The relationships between the different sites and NSBD traps are shown in Table 1. The NSBD traps were replaced with new ones containing fresh sugar bait (the same sugar bait components) every 2 weeks. Adult and larval mosquitoes were monitored every 2 weeks in each site using CDC light traps and larval pipette method (CI), respectively, during field trials, and the sampling method was the same to the monitoring before trials during April to June, with totally two trap-nights per site per month for adult mosquitoes and twice larval breeding sites monitoring per site per month for larval mosquitoes.

### 2.5 Statistical analysis

Statistical analyses were performed using the Statistical Package for the Social Sciences (SPSS, version 23.0). The adult mosquito and mosquito larval densities among the four villages before the field trials were compared using nonparametric tests to determine whether the baseline mosquito density among villages was similar. Generalized linear mixed models (GLMMs) were employed to assess differences in larval and adult mosquito density monitored in different test groups during trials. Mosquito larvae and adult density were used as dependent variables, NSBD traps (control group, NSBD-1, NSBD-2 and NSBD-3) and collection date as fixed independent variables (negative binomial regression model). The means and standard errors associated with GLMMs were calculated. The rates of decrease and the standard decrease in mosquito density (adult and larval mosquitoes) were calculated for all four villages before and after the trials.

The decrease rates of adult mosquito density = (the average density of adult mosquito before trials- the average density of adult mosquito after trials)/ the average density of adult mosquito before trials × 100%.

The standard decrease rates of adult mosquito density = (the decrease rates of adult mosquito density in the test group- the decrease rates of adult mosquito density in the control group)/ (1- the decrease rates of adult mosquito density in the control group) × 100%.

The decrease rates of mosquito larvae density = (the average density of mosquito larvae before trials- the average density of mosquito larvae after trials)/ the average density of mosquito larvae before trials × 100%.

The standard decrease rates of mosquito larvae density = (the decrease rates of mosquito larvae density in the test group- the decrease rates of mosquito larvae density in the control group)/ (1- the decrease rates of mosquito larvae density in the control group) × 100%.

## 3 Results

### 3.1 General information

Before the field trials, we monitored the density of adult and larval mosquitoes monthly for 3 months to confirm whether the density baseline among the four villages was comparable. A median of 58 (Interquartile Range, IQR: 46, 59), 63 (IQR: 56, 63.5), 42 (IQR: 39.5, 47) and 45 (IQR: 38.5, 50.5) of larval breeding sites per month were monitored in four sites, respectively. The densities are listed in Tables 2, 3. No statistically significant differences in adult and larval mosquitoes were found among the four villages before the trials (adults, *F* = 3.047, *p* = 0.384 > 0.05; larvae, *F* = 0.436, *p* = 0.933 > 0.05).

**Table 2:** The density of mosquito larvae^b^ among four sites before and after the trials.

Field trials	Month	Site 1^a^	Site 2	Site 3	Site 4
Before trials	April	11.54	12.24	10.81	9.38
	May	22.73	28.57	21.43	22.22
	June	57.50	40.63	53.85	57.14
After trials	Early July	27.03	22.22	10.00	27.78
	Late July	30.00	14.29	20.00	11.76
	Early August	25.45	44.44	23.68	10.00
	Late August	24.66	18.18	13.79	6.67
	Early September	/	/	/	/
	Late September	40.54	13.64	20.75	10.53
	Early October	29.73	21.05	20.83	13.33
	Late October	24.32	20.00	18.60	17.24
	Early November	20.29	12.50	11.43	9.68
	Late November	4.84	0.00	6.90	0.00
The decrease rates of mosquito larvae density (%)		−6.03	31.71	45.69	55.25
The standard decrease rates of mosquito larvae density (%)		/	35.59	48.78	57.80

a Site 1 is control group.

b The density of mosquito larvae was represented by Container index (CI).

**Table 3:** The density of mosquito adults^a^ among four sites before and after the trials.

Field trials	Month	Site 1	Site 2	Site 3	Site 4
Before trials	April	0	1	2	5
	May	8	3	8	12
	June	14	5	15	18
After trials	Early July	5	3	2	5
	Late July	8	2	2	4
	Early August	9	4	2	1
	Late August	12	4	4	2
	Early September	16	4	7	2
	Late September	18	7	11	2
	Early October	17	4	8	3
	Late October	12	1	3	2
	Early November	3	0	3	1
	Late November	1	0	1	0
The decrease rates of mosquito adult density (%)		-37.73	3.33	48.40	81.14
The standard decrease rates of mosquito adult density (%)		/	29.81	62.53	86.31

a The density of mosquito adults was represented by mosquitoes per CDC light trap per night.

### 3.2 Effects of NSBD traps on controlling mosquito larvae

A median of 62 (IQR: 42, 73), 54 (IQR: 45, 57), 58 (IQR: 53, 60) and 58 (IQR: 54, 60) of larval breeding sites per half-month were monitored in four sites, respectively. The density of mosquito larvae after 5 months of field trials is shown in Table 2. Natural fluctuations in the mosquito larval density were observed in the control group, with the mosquito larval density increased by 6.03% after the trials. However, different levels of larval density were found in the test groups (sites 2–4) after the trials. The rates of decrease in mosquito larval density among the three test villages were 31.71, 45.69, and 55.25%. After correction using the control group, the standard decrease rates of mosquito larvae density were 35.59, 48.78, and 57.80% at sites 2, 3, and 4, respectively (Table 2). Significant differences in larval mosquito densities were observed among the four sites after the trials (GLMM, *F* = 5.808, *p* < 0.05). Compared with the control group (site 1), both sites 3 and 4 showed significantly lower larval densities after the trials (GLMM, site 3 vs. site 1, *F* = 2.755, *p* < 0.05; site 4 vs. site 1, *F* = 4.083, *p* < 0.01). NSBD-3 traps at site 4 showed the best effect in controlling mosquito larvae though no statistical differences were found, with a sugar concentration (glucose) of sugar bait at 10%.

### 3.3 Effects of NSBD traps on controlling mosquito adults

**Figure 2:**
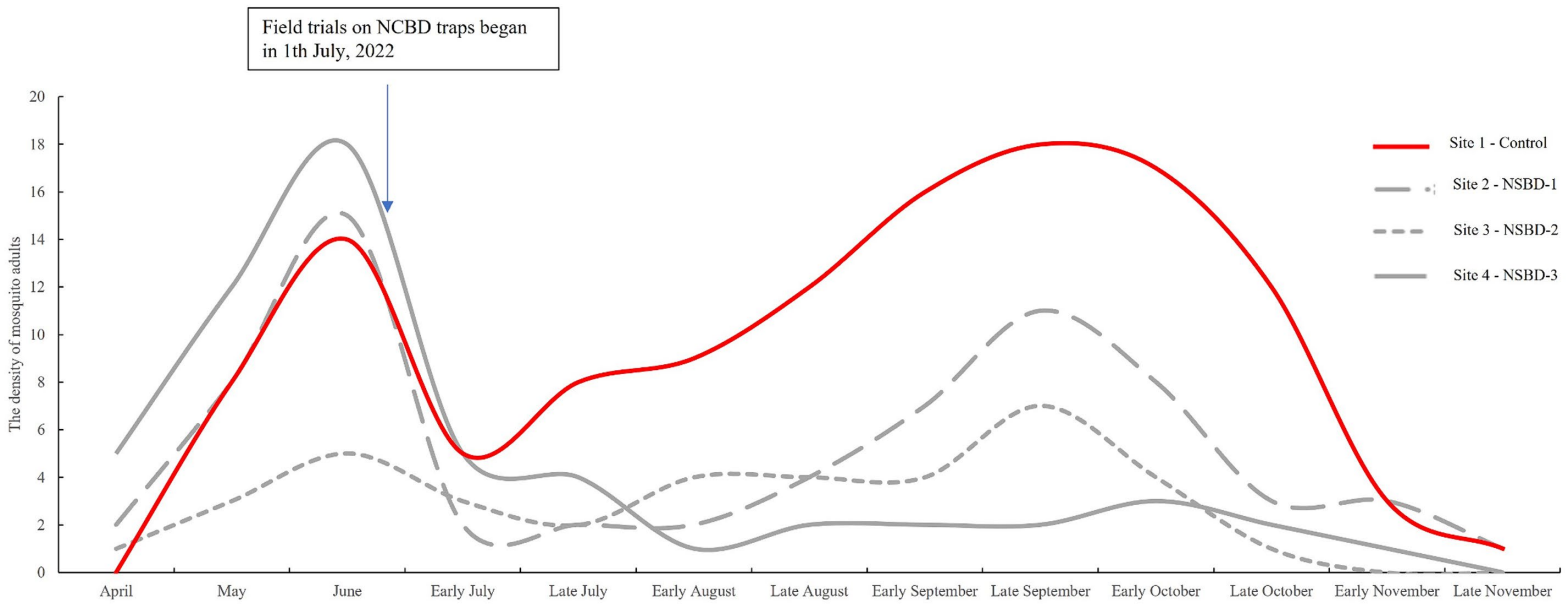
The trend of density changes in adult mosquitoes in four sites before and after trials.

During mosquito surveillance, a total of 202 adult mosquitoes had been captured by CDC light traps in site 2, 3, and 4. Five species were identified; *Cx.pipiens pallens* was the most abundant species (73.27%), followed by *Cx. tritaeniorhynchus* (10.89%) and *Ae. albopictus* (7.43%). The effect of NSBD traps on controlling adult mosquitoes was similar to that on larvae. The densities of adult mosquitoes in the four villages before and after the trials are shown in Table 3 and Figure 2. An increase in density was also observed in adult mosquitoes in the control group (site 1), with the density increased by 37.73% after the trials compared to that before. In the test groups (sites 2-site 4), the density of adult mosquitoes decreased significantly in all three villages. The rates of decrease in adult mosquito density among the three test villages were 3.33, 48.40, and 81.14%. After correction using the control group, the standard decrease rates of adult mosquito density were 29.81, 62.63, and 86.31% at sites 2, 3, and 4, respectively (Table 3). A significant difference in adult mosquito density was observed among the four sites after the trials (GLMM, *F* = 25.495, *p* < 0.01). Compared to the control group (site 1), sites 2, 3, and 4 showed significantly lower larval densities after the trials (GLMM, site 2 vs. site 1, *F* = 4.919, *p* < 0.01; site 3 vs. site 1, *F* = 6.213, *p* < 0.01; site 4 vs. site 1, *F* = 6.795, *p* < 0.01). NSBD-3 traps at site 4 showed statistically the best effect in controlling mosquito adults, with a sugar concentration (glucose) of sugar bait at 10%.

## 4 Discussion

Sugar bait has a mosquito-control history of over 60 years (8). Based on the results of previous studies, we developed a novel sugar bait device (NSBD) trap by combining sugar bait and physical adhesive capture technology to develop an effective and environmentally friendly method to control mosquitoes around human residents and prevent mosquito-borne diseases. This study evaluated the effects of NSBD traps with different sugar baits by controlling larval and adult mosquitoes in residential environments through field trials. The results showed that the NSBD traps could effectively reduce larval and adult mosquito densities, and the most suitable sugar solution concentration in the sugar bait was 10%.

In this study, four residential villages with similar geographical environments were selected. To better demonstrate the effectiveness of the NSBD, the basic mosquito densities in the four villages were assumed to be the same. Thus, 3 months of mosquito monitoring was conducted before the field trials, and no statistical differences were found in the densities of larval and adult mosquitoes among the four villages. NSBD traps were proven effective in controlling mosquitoes in residential environments, with the highest standard decrease rates of larval and adult mosquito densities of 57.80 and 86.31%, respectively. The mosquito densities at sites 3 (8% sugar solution concentration in sugar bait) and 4 (10% sugar solution concentration in sugar bait) were significantly lower than those at site 1 (control group). Similar effect had been found in some ATSBs with sugar concentrations ranging from 5 to 20% and insecticides on killing different species of mosquitoes in laboratory and field studies, which had reduced the density of mosquitoes by 52 to 96% after exposure to ATSBs (8, 12, 13).

The concept of insect baiting is not novel, it was first described in 77 AD in Historia Naturalis (14). Subsequently, an increasing number of insect-baiting attempts have been made to dissuade behavior and induce mortality (8). Sugar feeding is a basic biological habit of mosquitoes. Almost all adult mosquitoes feed on sugary meals to supplement their energy throughout their lives (12, 15–17). This behavior has always been used to control mosquitoes. In this research, sugar bait was combined with sticky black insect paper in a black bucket to lure and stick the mosquitoes, and it proved to be effective on controlling mosquitoes. Similar results had been found in MosHouse traps by Pattamaporn Kittayapong et al. After adding a sugar stick and sticky flags, its effect on capturing mosquitoes significantly increased (18). However, while NSBD traps attract and kill mosquitoes, ensuring that the provided sugar bait solution does not become a new breeding place for mosquito larvae is crucial, especially when we should use methods without chemical insecticides. Thus, many preliminary experiments have been conducted in the laboratory, and it has been shown that sugar solution concentrations above 5% could effectively prevent larval survival (unpublished data). After considering the luring effect and unsuitability for larval survival, a sugar bait with a sugar solution concentration of 8% was chosen as the baseline to explore the effect of controlling mosquitoes in real residential environments. Throughout the field trials, we found no live mosquito larvae in the NSBD traps. Black was chosen as the color of sticky insect papers and buckets because of the black preference characteristics of *Ae. albopictus*, which is one of the most advantageous mosquito populations in Zhejiang Province (19).

To control vector mosquitoes, several traps had been developed such as BG- Sentinel traps and backpack aspirators, which are chemically independent and effectively on collecting mosquitoes especially *Aedes* mosquitoes (20–22). However, they both require power source and are not cost-effective in mosquito collection (23, 24). So more economical traps had been developed to captured gravid mosquitoes such as Stick ovitraps, AedesTrap, both of which attract and capture gravid mosquitoes by creating suitable oviposition environments (23, 25). MosHouse trap was developed using odor created by hay infusion and oviposition environment to attract gravid and non-gravid mosquitoes (18). However, all of them use *Bacillus thuringiensis israelensis* (Bti) as substances to killing mosquito larvae, which is also one of insecticides and might induce drug resistance. The advantage of NSBD traps is low-cost, easy to use, without use of chemical insecticides and requires no power sources for its usage, it could be considered as an appropriate alternative trapping method to control mosquitoes around residential areas, then to reduce mosquito infestation and to prevent mosquito-borne diseases.

The limitation of this research is that within this investigation, only three concentration gradients of sugar solutions in the sugar bait were tested in the field trials; thus, caution should be exercised when extrapolating the results. However, according to the results of the laboratory experiments, the luring effect of sugar bait with a sugar solution concentration of 15% on *Ae. albopictus* and *C. quinquefasciatus* was much lower than that of 8%. Therefore, it can be inferred that the effect of sugar solution concentrations above 10% on mosquito control might not be better than that of concentrations below 10%. On the other hand, in the present study we focus more on the overall decrease in adult and larval mosquito density than on some specific mosquito species by considering that sugar bait could attract many mosquito species (8). And no comparison had been made on the control effect of NSBD traps on different mosquito species. In the future, more researches like the long-term efficacy of NSBD traps and mosquito species that are more likely to be controlled by NSBD traps, the variability in different environmental conditions and geographical locations would be conducted to clarify the application potential of NSBD.

In conclusion, a novel sugar bait device (NSBD) trap, developed by combining sugar bait and physical adhesive capture technology, can effectively control larval and adult mosquitoes in residential environments. Without the use of chemical insecticides, this could be an effective and environmentally friendly method for controlling mosquitoes. The most suitable sugar concentration for the sugar bait of the NCBD traps was 10%.

## Data availability statement

The original contributions presented in the study are included in the article/supplementary material, further inquiries can be directed to the corresponding author.

## Author contributions

YW: Investigation, Methodology, Writing – original draft, Writing – review & editing. CZ: Investigation, Writing – original draft. WF: Investigation, Resources, Writing – original draft. SF: Project administration, Resources, Writing – original draft. WD: Methodology, Resources, Writing – original draft. JW: Investigation, Writing – original draft. QL: Investigation, Methodology, Writing – original draft. TL: Investigation, Writing – original draft. ML: Investigation, Writing – original draft. ZG: Writing – original draft, Writing – review & editing.
